# Research Advances in How the cGAS-STING Pathway Controls the Cellular Inflammatory Response

**DOI:** 10.3389/fimmu.2020.00615

**Published:** 2020-04-28

**Authors:** Dongshan Wan, Wei Jiang, Junwei Hao

**Affiliations:** ^1^Department of Neurology, Xuanwu Hospital, Capital Medical University, Beijing, China; ^2^Department of Neurology, Tianjin Neurological Institute, Tianjin Medical University General Hospital, Tianjin, China

**Keywords:** inflammation, cGAS, STING, cGAMP, type I interferonopathies

## Abstract

Double-stranded DNA (dsDNA) sensor cyclic-GMP-AMP synthase (cGAS) along with the downstream stimulator of interferon genes (STING) acting as essential immune-surveillance mediators have become hot topics of research. The intrinsic function of the cGAS-STING pathway facilitates type-I interferon (IFN) inflammatory signaling responses and other cellular processes such as autophagy, cell survival, senescence. cGAS-STING pathway interplays with other innate immune pathways, by which it participates in regulating infection, inflammatory disease, and cancer. The therapeutic approaches targeting this pathway show promise for future translation into clinical applications. Here, we present a review of the important previous works and recent advances regarding the cGAS-STING pathway, and provide a comprehensive understanding of the modulatory pattern of the cGAS-STING pathway under multifarious pathologic states.

## Introduction

Pattern-recognition receptors (PRRs) serve as innate cellular sensors of danger signals, such as pathogen-associated molecular patterns (PAMPs) or danger-associated molecular patterns (DAMPs), and yield cellular-stress response. DNA molecules are vital genetic components within cells, which are compartmentalized restrictively into specific regions. The occasionally misplaced DNA is degraded rapidly by scavenger cells and extracellular or intracellular ribonucleases. Aberrant accumulation of DNA is relevant to tissue damage (1).

In 2008, several research teams discovered a new protein on the endoplasmic reticulum (ER) which can be activated by immune-stimulatory DNA (ISD) and initiate type-I interferon (IFN) responses, which was named “stimulator of interferon genes” (STING, also known as MITA, ERIS) (2–4). STING does not bind to DNA directly, and bacteria-derived cyclic di-guanylate monophosphate (c-dGMP) or cyclic di-adenosine monophosphate (c-dAMP) were confirmed to be ligands for STING (5, 6). Subsequently, it was found that some DNA sensors can facilitate STING activation, such as interferon gamma inducible protein 16 (IFI16) (7). However, STING activation could not be fully explained by the upstream factors/ligands that had been found. It was postulated that an unknown upstream regulator might be responsible for STING activation.

In 2013, Wu and Sun found that cyclic guanosine monophosphate-adenosine monophosphate (cGAMP) was a novel secondary messenger serving as a ligand of STING (8). Beside it, they purified a new protein named “cyclic-GMP-AMP synthase” (cGAS) that had cytosolic DNA-sensing ability and can synthesize cGAMP (8). Also, they found that the cGAS-cGAMP-STING pathway was indispensable for host anti-viral immunity (9). Their work filled in the gaps missing from upstream of STING.

Stimulator of interferon genes or cyclic-GMP-AMP synthase is expressed widely in a broad spectrum of cells including immune, non-immune, cancer cells (10). Mounting evidence has demonstrated that the cGAS-STING pathway is important for mediating cellular immune sensing, and shows particular responses pattern to the ISD distinguished from other nucleotide-sensing pathways. It is also regulated delicately by several molecules or feedback loops to maintain cellular homeostasis. Nevertheless, cGAS-cGAMP-STING pathway itself has distinctive or even opposing effects under different conditions.

In this review, we cover the roles of cGAS-STING pathway in cellular type-I IFN immune response, and several cellular processes including autophagy, survival and senescence. We also summarize the literature on intrinsic cellular mechanisms modulating cGAS-STING pathway as well as its cross-regulations with other DNA-sensing pathways. Moreover, the inflammation-modulation capacities of this pathway in infectious disease, inflammation and cancers have been elucidated too, and a pervasive pattern of this pathway has been described, which could provide a plausible explanation of the contradictory findings of studies. Finally, current or prospective therapeutic strategies targeting the pathway, and issues that need to be addressed in the future, are discussed.

## cGas Recognizes Cytosolic DNA and Produces cGamp

Cyclic-GMP-AMP synthase belongs to the structurally conserved cGAS/DncV-like nucleotidyltransferase (CD-Ntases) superfamily. The latter is expressed universally in prokaryotes and eukaryotes, and can use purines or pyrimidines selectively as substrates for the production of linear or cyclic di- or even tri-nucleotide compounds, which act as secondary intracellular messengers (11).

Cyclic-GMP-AMP synthase is distributed mainly in the cytosol (also nucleus in some specific conditions) (8). Generally speaking, cGAS is activated upon the recognition of B-type double-stranded DNA (dsDNA) without sequence-specificity but not A-type dsDNA or RNA (12, 13). Hybrid DNA:RNA or stem-like single-stranded DNA (ssDNA) are also low-affinity ligands for cGAS (14, 15). After binding with ligands, cGAS undergoes an allosteric structural change, and subsequently catalyzes its substrates guanosine triphosphate (GTP) and adenosine triphosphate (ATP) to produce a mixed phosphodiester-linked cyclic dinucleotide: G(2′–5′)pA(3′–5′)p cGAMP (abbreviated as 2′,3′-cGAMP or cGAMP) (16). cGAS also catalyze the synthesis of linear dinucleotides such as AMP-2′-ATP, GMP-2′-GTP, and AMP-2′-GTP as intermediate products (17).

**FIGURE 1:**
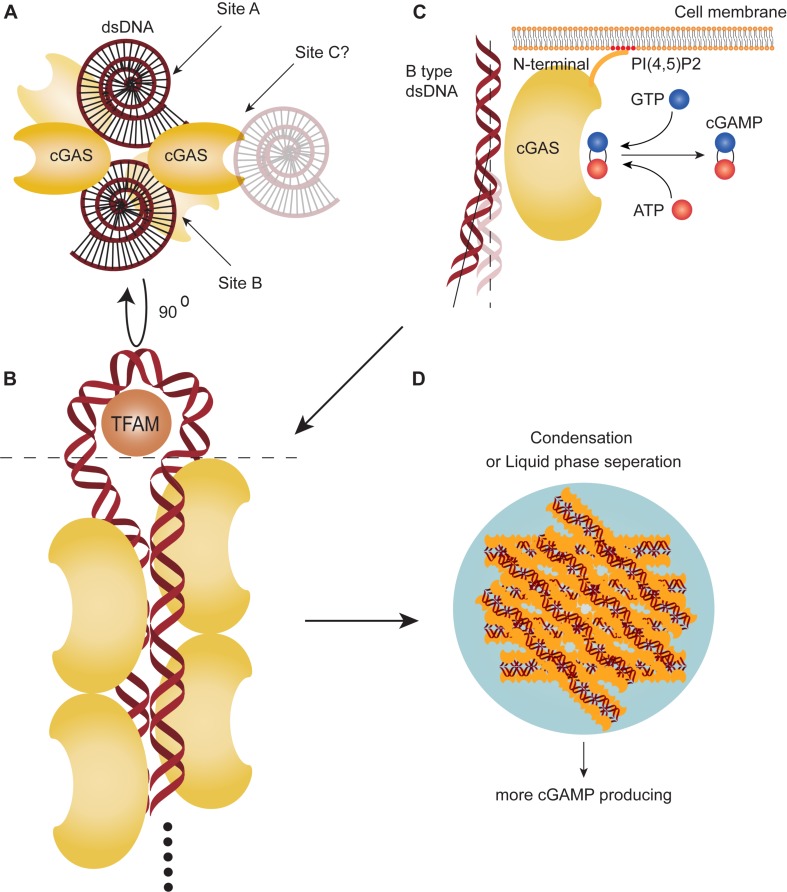
cGAS can recognize cytosolic DNA and produce cGAMP. **(A)** There are two DNA-binding sites on opposite sides of the catalytic pocket (site A,B) and a proposed DNA-binding site at the catalytic domain of cyclic-GMP-AMP synthesis (cGAS) (site C). **(B)** Multiple cGAS molecules can bind two double-stranded DNAs (dsDNA) to form a 2n:2 cGAS:dsDNA higher-ordered “ladder-like” oligomerization. Mitochondrial transcription factor A (TFAM) can bend the dsDNA into a U-shaped structure and promote polymerization. **(C)** cGAS can recognize B-type dsDNA. In humans, the cGAS DNA-interfacing residue of site A loosens the interaction of dsDNA to curve dsDNA away for more convenient binding with next adjacent cGAS. cGAS can catalyze GTP and ATP to synthesize cyclic guanosine monophosphate-adenosine monophosphate (cGAMP). The N-terminal tail binds to the cell membrane, associating with phosphatidylinositol 4,5-bisphosphate [PI (4,5)P2]. **(D)** Accumulation of cGAS-DNA complex goes through a liquid-phase separation and condenses into gel-like droplets.

There are two major dsDNA-binding sites on opposite sides of the catalytic pocket: A and B site. Site A is the primary contact surface for dsDNA, whereas site B is complementary, binding another dsDNA. It allows for cGAS to the formation of a 2:2 cGAS:dsDNA complex structure directed into two orientations with dsDNA at least 20 bp (18–20) (Figure 1A). Increased numbers of back-to-back dimers of cGAS hold the two dsDNA molecules together and permit successive recruitment of cGAS which, consequently, forms a 2n:2 cGAS:dsDNA higher-ordered “ladder-like” oligomerization, with cGAS arrayed “head to head/tail to tail” (19, 21). The DNA-binding protein HU, mitochondrial transcription factor A (TFAM), or bacterial high mobility group box 1 protein (HMGB1) can bend the dsDNA into a U-shaped structure and, thus, facilitate binding of cGAS dimers to the same strand as it travels in opposite directions (21) (Figure 1B). Human cGAS, unlike mouse cGAS, is prone to formation of this ladder-like network with long dsDNA, because of the human-specific residues K187 and L195. These two dsDNA-interfacing residues of site A loosen the interaction of dsDNA with cGAS, leading to dsDNA curving and allowing more convenient binding for the next adjacent cGAS (20, 21) (Figure 1C). Finally, accumulated cGAS-dsDNA complexes can go through a liquid-phase separation and condense into gel-like droplets as a reaction unit (Figure 1D). This conformation requires a sufficiently long dsDNA strand to form multivalent interaction positions, also requires the function of the N-terminal tail of cGAS and a recently discovered dsDNA-binding site in the catalytic domain of cGAS (site C) (22, 23). Meanwhile, the N-terminal tail of cGAS mediates cGAS localization onto the membrane by binding to phosphatidylinositol 4,5-bisphosphate (PI (4, 5) P2) and prevents liberation of cGAS and oligomerization, but can release cGAS during cell stress (24). The structure of cGAS determines long strand dsDNA (>500–1,000 bp) could potentially stimulate the enzyme activity and cGAMP production of cGAS (25). The ability of human cGAS to discriminate long dsDNA strands from shorter dsDNA may contribute to the specific sensing and recognition of the “danger DNA” of pathogens, necrotic cells or cancer cells rather than irrelevant shorter dsDNA, thereby enhancing the immunity against them specifically.

## Activation of The cGas-Sting Pathway

Double-stranded DNA is restricted into the nucleus or mitochondria and is rarely present in the cytoplasm. Extrinsic dsDNA from pathogens such as viruses, bacteria, transcellular vesicles or rupture of dying cells can be internalized into the cytosol in several diverse ways (26–28). These extrinsic dsDNA sources are engulfed by endosome through phagocytosis and digested immediately by DNaseII when fusing with lysosomes (29, 30). However, some escaping mechanisms under certain conditions could help protect them from being degraded. For example, antimicrobial peptide LL37 could efficiently transports self-DNA from endosome into cytosol of monocytes (28). Cell oxidative stress can lead to phagosomal acidification delay and probably release endosome context including dsDNA owing to increased membrane permeability (27, 31).

The intrinsic self-dsDNA can also be segregated inaccurately and released into the cytosol (32, 33). For example, genomic DNA (gDNA) injury as a result of genotoxic stress and DNA self-instability or replication errors leads to double-strand breaks (DSBs) and can be repaired by several ways (34). Impaired mediators of DNA-damage repair response mediators, such as ataxia telangiectasia mutated (ATM)-RAD3, poly ADP-ribose polymerase (PARP) and breast cancer1/2 (BRCA1/2) are associated with persisting DSBs and accumulation of cytosolic DNA (35–37). Extra-nuclear micronuclei formation during mitosis is a source of cytosolic dsDNA caused by DSBs (32, 38). Followed by homologous recombination repair of collapsed replication forks, DNA cleavage by methyl methanesulphonate (MMS) and ultraviolet-sensitive 81 (MUS81) also lead to cytosolic dsDNA presenting (39). Furthermore, manually Cre/loxP recombination technology can induce dsDNA damage during DNA cleavage, which results in the accumulation of cytoplasmic dsDNA (40). In normal cellular mitotic processes, chromosomal DNA can be exposed to the cytoplasm, while it is hard to bind and trigger cGAS (41).

**FIGURE 2:**
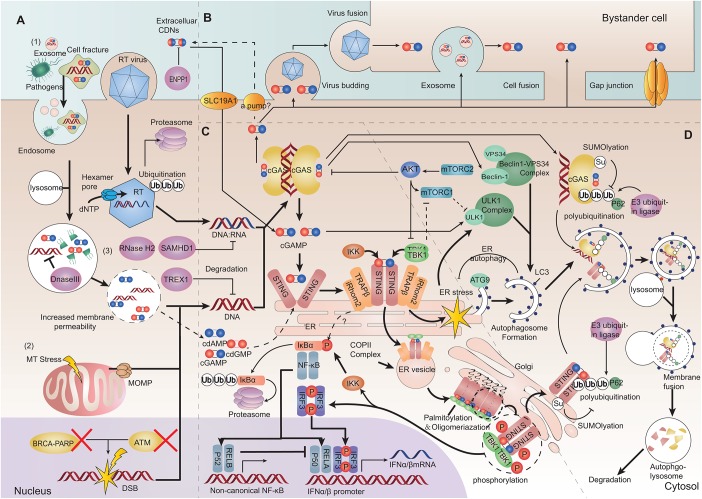
Depiction of the cGAS-STING pathway. **(A)** Cytoplasmic DNA challenge. (1) Extrinsic DNA source: extracellular DNA can be taken up into endosomes for digestion. Increased membrane permeability allows endolysosomal pathogenic DNA release into the cytosol. Viruses can release virons into the cytosol. The hexamer pore facilitates movement of nucleotides into the capsid. The latter can also be ubiquitinated and degraded. (2) Intrinsic DNA source: self-DNA from the nucleus with dysfunctional chromatin proteins can lead to chromatin DNA injury and gDNA presenting in the cytoplasm. Mitochondrial outer membrane permeabilization (MOMP) induced by mitochondrial stress can release oxidized mitochondrial DNA (mtDNA). (3) Three-prime repair exonuclease 1 (TREX1), DNase II, SAM domain, HD domain-containing protein 1 (SAMHD1) and RNaseH2 can restrict cytosolic DNA and DNA/RNA hybrids. cGAMP can be hydrolyzed by ectonucleotide pyrophosphatase/phosphodiesterase family member 1 (ENPP1). **(B)** cGAMP transferring between cells. cGAMP can be transferred between cells through viruses, membrane fusion, extracellular vesicles and cell gap junctions. Extracellular cyclic dinucleotides (CDNs) can transfer directly into cells by SLC19A1. CDNs (including cGAMP) might be exported by some ways. **(C)** Inflammatory signaling mediated by the cGAS-STING pathway. After sensing DNA, cGAS produces cGAMP and extracellular CDNs, promoting stimulator of interferon genes (STING) to undergo dimerization. STING can exit from the endoplasmic reticulum (ER), and be translocated from the ER to the ER-Golgi vesicle, and arrives at the Golgi. STING and TANK binding kinase 1 (TBK1) can be oligomerized and cluster at the Golgi. The STING-TBK1/IκB kinase (IKK) signalosome forms a scaffold to phosphorylate interferon regulatory factor 3 (IRF3) and inhibitor of NF-κBα (IκBα). Then, dimerized IRF3 and the activated canonical NF-κB p50/p65 complex can be translocated into the nucleus as transcription factors to promote transcription of type-I IFN. **(D)** Autophagy initiation and degradation. STING activation on ER triggers ER stress and mechanistic target of rapamycin complex1 (mTORC1) dysfunction. ER stress and mTORC1 dysfunction can stimulate the Unc-51 like autophagy activating kinase (ULK1) complex and beclin1-phosphatidylinositol 3-kinase catalytic subunit type 3 (PI3KC3) complex. Autophagy-related protein 9 (ATG9) and light chain 3 (LC3) are associated with genesis and elongation of the autophagosome. After autophagy initiation, cGAS-STING is ubiquitinated and binds with p62. Then, they are packaged into autophagosomes and terminally sorted to lysosomes (Bold arrows represent main signaling pathways, thinner arrows represent regulatory signaling pathways, and dashed arrows represent bypass or suspicious pathways).

In addition, mitochondrial DNA (mtDNA) is also a considerable ligand of cGAS and can be released into the cytosol under mitochondrial stress or dysfunction of proteins which participates in maintaining mitochondrial operations (33, 42, 43) (Figure 2A).

Cells have several types of nucleases to restrict cytosolic DNA to avoid cGAS activation. For example, three-prime repair exonuclease 1 (TREX1 also known as DNaseIII) is a cytosolic DNA exonuclease which removes unprotected dsDNA from the cytosol (44). RNaseH2 locates to the nucleus and specifically degrades the RNA in RNA:DNA hybrids participating in DNA replication (45). DNaseII is a lysosomal DNase which degrades undigested DNA in endosomes or autophagosomes to prevent their entry into the cytoplasm (30). SAM domain and HD domain-containing protein 1 (SAMHD1) is characterized as a dNTPase and restricts reverse transcription of the RNA virus (46, 47). SAMHD1 can also stimulate the exonuclease (but not the endonuclease) activity of MRE11 to degrade nascent ssDNA, and start DNA-repair responses at stalled replication forks (48). Depletion of SAMHD1 leads to the cleaving of nascent ssDNA by the activity of MRE11 endonuclease and cytosolic translocation of gDNA (48). Deficiency of any of these nucleases can lead to accumulation of self-DNA in the cytoplasm, thereby activating the cGAS-STING pathway against DNA molecules (30) (Figure 2A).

## cGamp Can Transfer Between Cells and Activate Sting

The production of asymmetrically linked 2′,3′-cGAMP catalyzed by cGAS has the highest affinity for STING to promotes STING dimerization (49, 50). cGAMP as a second messenger can be also transferred among cells in several ways to pass danger signaling of cytosolic DNA. Intercellular gap junction consists of two docking hexamer channels formed by different connexins, which allows many small molecules, including cGAMP, to pass bi-directionally through cells. And intercellular transfer of cGAMP through gap junction is largely dependent on connexin 43 (51–53). Additionally, cGAMP can be packaged into virons and pre-notify newly infected cells (54, 55). Cell fusion is a distinct manner for intracellular transmission of the human immunodeficiency virus (HIV); cGAMP also enter membrane-fused bystander cells in this way (56). Extracellular vesicles such as exosomes can contain cGAMP along with viral DNA, host gDNA or mtDNA, and mediate cells communication (57, 58). There were no evidences that cGAMP could be pumped out to extracellular space by a channel/transporter. However, it was found that SLC19A1 can transmit cyclic dinucleotides (CDNs) into cell plasma (59, 60). Notably, ectonucleotide pyrophosphatase/phosphodiesterase family member 1 (ENPP-1) can degrade extracellular cGAMP (61) (Figure 2B). Besides triggering STING, these exogenous cGAMP can directly bind to cGAS and prompt its activation as well (62).

After binding to cGAMP, the “lid” region of the STING dimer undergoes a conformational change that converts STING from an inactive “open” formation to an active “closed” formation. Following that, the STING dimer translocates from the ER to perinuclear ER-Golgi intermediate compartment (ERGIC) vesicles, finally arriving at the Golgi to form punctuate structures with downstream molecules (2, 63). ER-retention of STING caused by mutations results in reduced IFN signaling (64, 65). The translocon-associated protein β (TRAPβ) recruited by inactive rhomboid protein 2 (iRhom2) initially forms the TRAP translocon complex that mediates STING exit from the ER (2, 66). They both assist cytoplasmic coat protein complex-II (COPII) to drive ER-vesicle formation and carry the STING complex to the Golgi (67, 68).

Trafficking STING can bind directly to and be phosphorylated by TANK binding kinase 1 (TBK1) dimer or IκB kinase (IKK) complex (3, 69, 70). The C-terminal tail (CTT) of STING is a linear unfolded segment, which determines the optimization of combination specificity. STING CTT in mammals tends to bind TBK1, whereas in fish it tends to activate nuclear factor-kappaB (NF-κB) signal (71). The STING phosphorylation site Ser366 in the CTT cannot reach the kinase-domain active site of its directly bound TBK1, instead can reach the kinase-domain active site of the next adjacent TBK1 binding with another STING and be phosphorylated, while TBK1 phosphorylate each other (72, 73). Hence, STING and TBK1 can aggregate on the Golgi to form the STING signalosome. Clustering STING undergoes palmitoylation and full activation (74). It is also possible for STING-IKK to cluster and form the STING signalosome in this manner. The STING-TBK1/IKK signalosome produces a scaffold to phosphorylate interferon regulatory factor 3 (IRF3) or inhibitor of NF-κBα (IκBα). Activated IRF3 undergo dimerization (70). The activation of IκBα leads to its polyubiquitination and degradation by the proteosome, thereby eliminating its inhibition of NF-κB. There is also evidence suggesting that NF-κB activation might not require STING trafficking from the ER (75). Then, the dimerized IRF3 or activated NF-κB p50/p65 (p65 is also known as RelA) complex are translocated into the nucleus as transcription factors and bind to the promoter of type-I IFN to aid the transcription of type-I IFN (2, 3, 70). Meanwhile, activation of NF-κB p52/RelB can prevent recruitment of p65 and inhibit the p50/p65 signal (76) (Figure 2C).

## Sting Downstream Signaling Promotes Ifnα/β Expression and Autophagy

Expressed type-I IFN can propagate among cells in paracrine or autocrine manners. The binding of IFNα/β with its receptor triggers janus kinase (JAK) and signal transducer and activator of transcription (STAT) pathways, then induce transcription of type-I IFN-stimulated genes (ISGs), which have IFN-sensitive response elements (ISREs) in their 5′-untranslated regions (UTRs) (77). IRF3 can also bind partially to several ISREs alone (78). Herein, the expression of some ISGs including interferon-induced protein with tetratricopeptide repeats (IFIT) and pro-inflammatory cytokines such as tumor necrosis factor α (TNFα), interleukin (IL)-6, C-X-C motif chemokine ligand 10 (CXCL10) and C-C motif chemokine ligand 5 (CCL5) is increased remarkably by the cGAS-STING pathway (79). Furthermore, cGAS and STING are both ISGs, suggesting a positive feedback loop in spreading of the IFN signal (80, 81).

Stimulator of interferon genes activation on the ER also triggers an ER stress response with an “unfolded protein response (UPR) motif” on the C-terminus of STING, which leads to and ER stress-mediated autophagy (82, 83). STING-TBK1 activation and ER stress also induce mechanistic target of rapamycin complex 1 (mTORC1) dysfunction (84). ER stress or reduced mTORC1 signaling activates Unc-51-likeautophagy activating kinase (ULK1) complex and the Beclin-1-class III phosphatylinositol 3-kinase (PI3KC3 also known as VPS34) complex, which promotes initiation of the classical autophagy path (85). cGAS can also interact directly with the autophagy protein beclin-1-PI3KC3 complex and trigger autophagy (86). Furthermore, cGAS-dsDNA polymer can form a liquid-phase condensate (as mentioned above), which could theoretically be an initiator of autophagy (87). After autophagy initiation, autophagy-related protein 9 (ATG9) undertakes the genesis of the autophagosome along with light chain3 (LC3) undergoing lipidation, thereby resulting in elongation of the autophagosome (88). LC3 can also be recruited directly by ERGIC-loading STING and bypass the classical autophagy pathway (68, 89).

cGAS-STING-mediated autophagy can spread to the whole cell and help the elimination of intracellular microorganisms, subcellular organelles or misfolded proteins, as well as the ER itself that loads the STING signalosome (90–92) (Figure 2D). cGAS-STING-mediated autophagy is also indispensable for removing cytosolic DNA and inflammatory signaling factors to restrict the inflammatory response raised by the pathway itself (93). Excessive signaling of the autophagy cascade can lead to irreversible apoptosis termed “autophagic cell death” (94). Consequently, oligomerized cGAS or STING undergoes ubiquitination and is packaged into autophagosomes with the help of p62, to be terminally sorted into lysosomes (79, 83, 95, 96). cGAS or STING is digested immediately in the autophagolysosome after transient activation of downstream signaling (68, 79, 83, 89). Autophagy functions as a negative feedback loop which ensures transient cGAS-STING signaling and avoids consistent over-reaction of the pathway. Thus, impairment of autophagy may give rise to destructive inflammatory diseases (31).

## Regulation of the cGas-Sting Pathway

We cataloged factors in the literature that could potentially up- or down-regulate expression of cGAS/cGAMP/STING in pre-translational or post-translational stages (Tables 1, 2). The regulatory mechanisms of TBK1, IRF, and NF-κB in signaling pathways associated with expression of type-I IFN are outside the scope of this review.

**TABLE 1:** Factors promoting cGAS-STING pathway.

Functions	Factors	Targets	Mechanisms	References
E3 ubiquitination	TRIM56	cGAS	Monoubiquitination at K335	(277)
		STING	K63-linked polyubiquitination at K150	(278)
	RNF185	cGAS	K27-linked polyubiquitination at K173/384	(279)
	RNF26	STING	K11-linked polyubiquitination at K150	(280)
	AMFR INSIG1	STING	K27-linked polyubiquitination at K137/150/224/236	(281)
	MUL1	STING	K63-linked ubiquitination of K224	(75)
Deubiquitination	USP14	cGAS	Cleaving K48-linked polyubiquitin chain at K414	(282)
	USP21	STING	Cleaving K27/63-linked polyubiquitin chain	(283)
	USP20	STING	Cleaving K27/63-linked polyubiquitin chain	(284)
	CYLD	STING	Cleaving K48-linked polyubiquitination	(285)
	EIF3S5	STING	Cleaving K48-linked polyubiquitination	(66)
E3 SUMOylation	TRIM38	cGAS	Sumoylating at K231/479 to prevent polyubiquitination	(286)
		STING	Sumoylating of STING at K338 to prevent polyubiquitination	
De-SUMOylation	SENP7	cGAS	Cleaving SUMO on the K335/372/382	(287)
Directly interacting	G3BP1	cGAS	Supporting formation of large cGAS complexes	(288)
	zinc ion	cGAS	Promoting cGAS liquid phase condensation	(22)
	ZCCHC3	cGAS	Enhancing the binding of cGAS to dsDNA	(289)
	Manganese ion	cGAS	Enhancing the binding of cGAS to dsDNA	(290)
		STING	Enhancing cGAMP-STING binding affinity	
	TMEM203	STING	Promoting activation	(291)
	ZDHHC1	STING	Promoting dimerization	(292)
	TMED2 or TMED10	STING	Promoting recruitment of STING into the COPII Complex for trafficking	(67)
	IFIT3	STING- TBK1	Promoting STING-TBK1 binding	(293)
	S6K1	STING- TBK1	Forming of a tripartite S6K1-STING-TBK1	(294)
	GSK3b	STING-TBK1	Promoting TBK1 autophosphorylation at Ser172 and promoting its binding to STING	(295, 296)
Processing body-associated protein	LSm14A	STING	Processing STING nuclear mRNA precursor and maintaining mRNA level	(297)

*TRIM, tripartite motif-containing; UBXNs, ubiquitin regulatory X domain-containing proteins; RNF, RING finger domain; AMFR, autocrine motility factor receptor; INSIG1, insulin-induced gene 1; MUL1, mitochondrial E3 ubiquitin protein ligase 1; USP, ubiquitin-specific protease; CYLD, cylindromatosis; EIF3S5, eukaryotic translation initiation factor 3S5; iRhom2, inactive rhomboid protein 2; SUMO, small Ubiquitin-like Modifier; SENP7, sentrin/SUMO-specific protease 7; G3BP1, activating protein SH3 domain–binding protein 1; ZCCHC3, CCHC-type zinc-finger protein 3; TMEM, transmembrane protein; TMED, transmembrane p24 trafficking protein 1; TRAPβ, translocon-associated protein β; IFIT3, interferon-induced protein with tetratricopeptide repeats 3; S6K1, ribosomal protein S6 kinase 1; mTOR, mechanistic target of rapamycin; GSK3b, glycogen synthasekinase 3b; CREB, c-AMP-response element binding protein.*

**TABLE 2:** Factors inhibiting cGAS-STING pathway.

Functions	Factors	Targets	Mechanisms	References
E3 ubiquitination	TRIM29	STING	K48-linked ubiquitination at K370	(298)
	TRIM30α	STING	K48-linked ubiquitination at K275	(299)
	RNF5	STING	K48-linked ubiquitination at K150	(300)
Deubiquitinating	USP13	STING	K27-linked deubiquitination at ?	(301)
Desumoylation	Senp2	cGAS	Desumoylating at K231/479 allowing K48-linked ubiquitination	(286)
		STING	Desumoylating at K338 allowing K48-linked ubiquitination	
Directly interacting	Cia-cGAS	cGAS	A circular RNA harboring a stronger affinity than dsDNA	(135)
	OASL	cGAS	Ubiquitin-like domain inhibiting cGAS enzymatic activation	(302, 303)
	AKT1	cGAS	Phosphorylating the S291/S305 of the enzymatic domain	(245)
		STING- TBK1	Directly phosphorylating TBK1 preventing the TBK1–STING association	(244)
	RECON	STING/NF-κB	Competitively binding with CDNs	(304)
	STIM1	STING	Retaining STING in the ER membrane without activation	(305)
	Nitro-fatty acids	STING	Nitro-alkylation inhibiting palmitoylation	(306)
	NLRC3	STING/TBK1	Blocking STING-TBK1 interaction	(307)
	PPM1A	STING/TBK1	Dephosphorylating	(308)
	PTPN1/2	STING	Dephosphorylating at Y245	(309)
	NLRX1	STING- TBK1	Blocking STING-TBK1 interacting	(310, 311)
	SOX2	STING	Promoting autophagy-dependent degradation	(312)
MicroRNA	miRNA25/93	cGAS	Inhibiting expression of NCOA3 which promotes transcription	(313)
transcription factor	Nrf2	STING	Decreasing mRNA stability	(314)
Histone H3K4 lysine demethylase	KDM5	STING	Suppressing the expression	(315)
Phosphodiesterase	ENPP1	cGAMP	Hydrolyzing cGAMP	(61)

*Senp2, sentrin/SUMO-specific protease 7; TRIM, tripartite motif-containing; RNF5, RING finger domain; USP, ubiquitin-specificprotease; OASL, 2′-5′-oligoadenylate synthase; mTORC2, mechanistic target of rapamycin complex 2; DDX58, DExD/H-box helicase 58; STIM1, stromal interaction molecule 1; NLRC3, NOD-like receptor family CARD domain containing; PPM1A, protein phosphatase 1A; PTPN, protein tyrosine phosphatases non-receptor type; NLRX1, NLR family member X1; SOX2, SRY-box 2; Nrf2, nuclear factor (erythroid-derived 2)-like 2; KDM5, lysine-specific demethylase 5; ENPP1, ectonucleotide pyrophosphatase/phosphodiesterase family member 1; NCOA 3, nuclear receptor co-activator 3; TLR, Toll-like receptors.*

## Cross-Regulation of the cGas-Sting Pathway With Other DNA-Sensing Pathways

**FIGURE 3:**
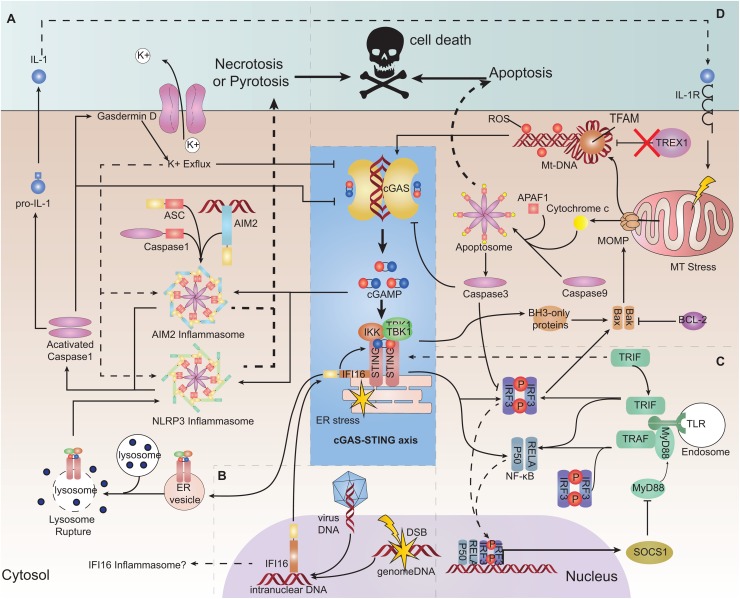
Interaction of the cGAS-STING pathway with other DNA-sensing pathways and its role in cell survival. **(A)** Absent in melanoma2 (AIM2) pathway and pyroptosis and necroptosis. AIM2 can be triggered by cGAMP and form an inflammasome, consequently triggering interleukin (IL)-1 production and pyroptosis. Stimulator of interferon genes (STING) trafficking to the lysosome ruptures the lysosome membrane, resulting in K^+^ efflux and activation of the NLRP3 inflammasome, leading to pyroptosis. Cyclic-GMP-AMP synthase (cGAS) and interferon regulatory factor 3 (IRF3) can be a target for caspase-1 cleaving. Gasdermin D can lead to K^+^ efflux and inhibition of cGAS. **(B)** Interferon gamma inducible protein 16 (IFI16). IFI16 can be transported to the cytoplasm to help to recruit STING and TANK binding kinase 1 (TBK1). IFI16 as a PYHIN family protein may form the inflammasome only in theory. **(C)** Toll-like receptor (TLR) pathway. TIR domain-containing adaptor-inducing IFNβ (TRIF) may be responsible for helping the dimerization of STING. STING signaling can induce suppressor of cytokine signaling 1 (SOCS1) expression, which negatively regulates MyD88 activity. **(D)** Apoptosis. Mitochondrial outer membrane permeabilization (MOMP) formed by BAX/BAK induced by mitochondrial stress can release oxidized mitochondrial DNA (mtDNA) and cytochrome c into the cytosol. Oxidized mtDNA is a suitable ligand for cGAS recognition and is resistant to DNaseIII (TREX1) degradation. Cytochrome c binds to apoptotic protease-activating factor 1 (APAF1) and initiates the formation of an apoptosome cooperatively with caspase-9 to activate caspase-3, which can induce apoptosis. Caspase-3 can cleave cGAS.

PYHIN family member absent in melanoma 2 (AIM2) is a cytoplasmic dsDNA sensor. It can recruit apoptosis-associated speck-like protein containing a CARD (ASC) by its PYHIN domain and form the AIM2 inflammasome. The inflammasome activates caspase-1, which activates IL-1 and trigger pyroptosis (97). The AIM2 pathway could counteract the cGAS-STING pathway (98). First, cGAS is a target for caspase-1 cleavage (99). Second, gasdermin D activated by caspase-1 can lead to potassium ion (K^+^) efflux which inhibits cGAS (100). Conversely, the cGAS-STING pathway can trigger the AIM2 inflammasome or NLR family pyrin domain containing 3 (NLRP3) by several means, and the process lags behind canonical IFN signaling (96, 101). In this way, the inhibitory nucleic-acid sensor NLR family CARD domain containing 3 (NLRC3) can counteract STING by binding and occupying it, but viral DNA as a possible NLRC3 ligand can reverse its occupation of STING (102) (Figure 3A).

Another PYHIN family member, IFI16, is a DNA sensor located in the nucleus. IFI16 can bind to viral DNA sequences or damaged chromatin DNA and be translocated to the cytoplasm to recruit STING cooperatively with TNF receptor associated factor 6 (TRAF6) and p53 (103, 104). Several studies have shown that IFI16 (which can stimulate the phosphorylation and recruitment of STING and TBK1) is required for the full response of STING (105, 106) (Figure 3B). Conversely, cGAS can partially enter the nucleus and interact with IFI16 to promote its stability (107). Therefore, it is inferred that during viral infection, IFI16 can facilitate recognition of decapsidated viral DNA in the nucleus, while cGAS in the cytoplasm engages with viral gene transcription products (104, 108). However, STING signaling can trigger IFI16 degradation by tripartite motif-containing 21 (TRIM21) ubiquitination (109).

TLR is also an important PRR for multiple PAMPs (110). TIR domain-containing adaptor-inducing IFNβ (TRIF) is downstream of several subtypes of TLRs (including TLR3). TRIF may be responsible for interacting with STING and helping the dimerization of STING (111). During viral infection, the TLR9-myeloid differentiation primary response 88 (MYd88)-IRF3/7 pathway is necessary for mouse monocytes recruitment to lymph nodes, whereas the STING pathway is necessary for local production of type-I IFN (112). However, STING signaling can induce suppressor of cytokine signaling1 (SOCS1) expression, which can negatively regulate MyD88 activity (113) (Figure 3C).

## cGas-Sting Pathway in Cell Survival

Oxidized mtDNA can be released into the cytoplasm during cell stress elicited by hypoxia, viral infection and mitochondrial damage, etc.; oxidized mtDNA is resistant to degradation by the cytosolic nuclease TREX1 (114). In addition, mtDNA accompanied with TFAM (a mtDNA-binding protein that can bend mtDNA) is also a reasonable target for recognition by cGAS (21, 33). However, during regulated cell death (as represented by apoptosis), it undergoes mtDNA release but has certain mechanisms to ensure a minimal cGAS-STING-mediated immune response. Mitochondrial outer membrane permeabilization (MOMP) activation, which is executed by BCL-2-associated X protein (BAX) and BCL-2 antagonist or killer (BAK), is a highly controlled conserved process in regulated cell death. BAK and BAX activated by apoptosis signals cooperatively form a pore-like conformation on the mitochondrial outer membrane, leading to a permeability change of outer and also inner membranes (115, 116). Consequently, the mitochondrial matrix, including cytochrome C and oxidized mtDNA-TFAM, is released into the cytoplasm (115, 117). Cytochrome C binds to apoptotic protease-activating factor 1 (APAF1) and initiates the formation of the apoptosome cooperatively with caspase-9, which further triggers the intrinsic apoptosis program (117). *In vivo* and *in vitro* studies have shown that an absence of caspase-9 is associated with greater release of type-I IFN (43, 117). This occurs because caspase-9 and its downstream caspase-3 can cleave cGAS and IRF3 to restrain deleterious inflammation (118) (Figure 3D).

The cGAS-STING pathway can also initiate programmed cell death. Activation of STING enhances phosphorylation and activation of receptor interacting serine/threonine kinase 3 (RIP3) and mixed lineage kinase domain-like pseudokinase (MLKL). Proapoptotic BCL2 binding component 3 (PUMA), a member of BH3-only family, is subsequently activated in a RIP3/MLKL-dependent manner, which promotes leakage of mtDNA by MOMP (119, 120). Activated IRF3 can bind directly to BAX to form IRF3/BAX complex and induce apoptosis (47). Excessive cGAS-STING-mediated autophagy signaling can cause “autophagic cell death” and prevent malignant transformation of cells through DNA damage (94, 121). STING trafficking to the lysosome can broaden permeabilization of the lysosome membrane, thereby rupturing the lysosome and releasing its contents, resulting in “lysosomal cell death (LCD)”. LCD further triggers K^+^ efflux and NLRP3 activation, ultimately resulting in pyroptosis (96, 101) (Figure 3D). Moreover, stimulating STING-dependent type-I IFN and TNFα signals simultaneously can lead to necroptosis of tumor cells (122, 123).

## cGas-Sting Pathway in Cell Senescence

Cell senescence is recognized as a permanent arrest of the cell cycle, and is common in aging, immunity, ontogenesis and infectious defense (124). It lacks a specific biomarker but can be identified by the expression of several anti-proliferative molecules (representatively Rb-p16 andp53-p21 pathway) (125). During senescence, changes in the nuclear structure and loss of the nuclear lamina protein disrupt the integrity of the nuclear envelope, leading ultimately to DNA damage and cytoplasmic chromatin fragments (126). Cellular senescence can be accelerated by accumulation of cytoplasmic chromatin in turn (127). These senescent cells produce the senescence-associated secretory phenotype (SASP), which shapes an inflammatory microenvironment (128). The cGAS-STING pathway has been reported to be involved in the recognition of cytoplasmic chromatin fragments from senescence-related DNA damage, and mediate the expression of SASP genes (129–132). Along with these actions, the expression of TREX1 and DNaseII is inhibited by DNA damage through the inhibition of E2F/DP (a potential transcription factor of TREX1 and DNaseII) (130).

For hematopoietic stem cells (HSCs), DNA damage can promote excessive secretion of type-I IFN in the HSC *niche* and activate p53 pathway, both of which can lead to long-term senescence and exhaustion of HSCs (133, 134). HSCs expressing a circular RNA named “cia-cGAS” in the nucleus, however, are protected from this exhaustion as a result of cia-cGAS having stronger affinity than that of self-DNA, which prevents it from being sensed (135). It implied a novel target to manipulate the immune environment in bone marrow and help for finding treatment approaches for hematopoiesis-based diseases, such as aplastic anemia. Utilizing cellular senescence to restrain tumor growth is discussed below.

## cGas-Sting Pathway in Infection

cGAS-STING signaling has an essential role in defense against a broad spectrum of intracellular DNA and RNA viruses (9, 26, 50). HIV is a typical RNA retrovirus: there is neither dsDNA in its genome, nor production of nucleic acids (50). Nevertheless, cGAS can detect the presence of HIV. RNA:DNA hybrids synthesized during reverse transcription that can be sensed by cGAS explain (at least in part) this phenomenon (14). cGAS may be triggered by endogenous DNA broken and released during HIV infection as well theoretically. However, some studies found the new mechanisms. The early reverse-transcription production of HIV-1 can flank short stem loops with paired base, which lead to the production of Y-type DNA containing unpaired guanosines that can activate cGAS well (15). Moreover, nucleolus protein non-POU domain-containing octamer-binding protein (NONO), as a sensor of capsid components of HIV, can help cGAS to be translocated to the nucleus and assist cGAS to sense HIV DNA accompanied by polyglutamine-binding protein 1 (PQBP1) (136, 137). The assistance proffered by NONO in assisting cGAS to sense DNA is also associated with its role in constructing a ribonuclear complex with DNA-dependent protein kinase (DNA-PK) subunits around hexamethylene bis-acetamide-inducible protein1 (HEXIM1), termed as “HEXIM1- DNA-PK - paraspeckle components-ribonuclear protein complex (HDP-RNP),” which also has a role in repair of DNA damage and transduction of genotoxic signals (138). This complex is also required to accompany cGAS-PQBP1 in sensing DNA virus, such as Kaposi’s sarcoma-associated herpes virus (139). In addition, during virus infection, STING activation can lead to global suppression of translation in cells, which restricts viral replication (140).

Compared with HIV-1, HIV-2 is less infective because it can infect dendritic cells (DCs) and elicit an anti-virus immune response. As a result, HIV-2 can cross-protect against HIV-1 (141). This phenomenon has been attributed to the fact that HIV-2 (instead of HIV-1) can encode protein Vpx, which overcomes the SAMHD1 restriction of dNTP in DCs (46, 142). HIV-1 can infect DCs *via* Vpx presentation, nevertheless, HIV-1 still cannot be fully sensed and induce an efficient immune response owing to certain escape mechanisms. Whether it is HIV-1 or HIV-2, a completely robust IFN response is required at pre- and post-integration sensing stages (143). cGAS in DCs can detect reverse-transcribed cDNA of HIV-2 before and after integration, whereas HIV-1 sensing is after genome integration owing to its capsid protection (144, 145). It was suggested that during initial infection by HIV-1, nucleotides are recruited into the intact capsid through the hexamer pores on the HIV-1 capsid. Therefore, the capsid-coated HIV-1 virus prevents the encapsidated reverse-transcription production from being sensed by the cytosolic nucleic-acid sensors (146). HIV-1 capsids can be ubiquitinated and then degraded by the host E3 ubiquitin ligase function of TRIM5, which leads to detection of viral DNA, meanwhile HIV-1 could use some host protein like cyclophilins to evade the sensing (147, 148) (Figure 2A).

Similarly, other viruses also have evasion mechanisms to escape cGAS-STING pathway surveillance (Table 3). Therefore, identifying and preventing such viral-evasion factors could be a viable means to design novel anti-viral drugs.

**TABLE 3:** Virus productions associated with evasion from cGAS-STING pathway.

Virus	Proteins	Targets	Functions	References
HSV-1	UL41	cGAS	Selectively degrading cGAS mRNA	(316)
	VP22	cGAS	Blocking cGAS enzymatic activity	(317)
	UL37	cGAS	Deamidation	(318)
	UL46	STING	NA	(319)
	γ1 34. 5 Protein	STING	Blocking translocation of STING	(320)
CMV	UL31	cGAS	Disassociating DNA from cGAS	(321)
	UL83	cGAS	Inhibiting cGAMP producing	(322)
	UL48	STING	Cleaving K63-linked ubiquitin of STING	(323)
	UL82	STING	Disrupting the STING-iRhom2-TRAPβ translocation complex	(324)
	m152	STING	Blocking STING trafficking to Golgi	(325)
	US9	STING	Disrupting STING oligomerization	(326)
KSHV	ORF52	cGAS	Inhibiting cGAS enzymatic activity and interfering interaction between the HDP-RNP and cGAS	(139, 327)
	LANA	cGAS	Blocking cGAS enzymatic activity	(328)
	vIRF1	STING	Blocking TBK1–STING interaction	(329)
Coronavirus	PLPro	STING	Inducing an incomplete autophagy and degration of STING and blocking STING-TRAF3-TBK1 complex formation and ubiquitination of STING	(330)
Poxvirus	F17	cGAS	Disrupting mTOR pathway to promote cGAS degradationa and blocking STING trafficking	(331)
		STING	Disrupting mTOR pathway to block STING trafficking	
	Poxins	cGAMP	Hydrolyzing cGAMP	(332)
Zika virus	NS1	cGAS	Cleaving K11-linked polyubiquitin chains from caspase-1 to promote the cleaving of cGAS	(333)
	NS2B3	STING	Promoting degration of STING	(334)
Dengue virus	NS2B	cGAS	Promoting autophagy-dependent cGAS degradation	(335)
HPV	E7	STING	Inhibiting STING with LXCXE motif	(336)
adenovirus	E1A	STING	Inhibiting STING with LXCXE motif	(336)
Influenza A virus	FP	STING	Blocking STING dimerization	(337)
HCV	NS4B	STING	Blocking interaction between STING and TBK1	(338, 339)
HBV	Pol	STING	Decreasing K63-linked polyubiquitination of STING	(340)
HIV	vpx	STING- NF-κB	Selectively suppressing STING-mediated NF-κB signaling	(341)

*HSV, herpes simplex virus; CMV, cytomegalovirus; iRhom2, inactive rhomboid protein 2; TRAPβ, translocon-associated protein β; KSHV, kaposi’s sarcoma-associated herpes virus; HDP-RNP, HEXIM1-DNA-PK-paraspecklecomponents-ribonuclear protein complex; LANA, latency-associated nuclear antigen; vIRF1, viral interferon regulatory factor 1; PLPro, papain-like protease; LC3, light chain3; mTOR, mechanistic target of rapamycin; TRAF3, TNF receptor associated factor 3; HCV, hepatitis c virus; HPV, human papilloma virus; HBV, hepatitis B virus; HIV, human immunodeficiency virus; NF-κB, nuclear factor-*κ*B.*

cGAS-STING pathway is responsible to protect against intracellular or extracellular bacterial infection (especially intracellular infections). CDNs (e.g., c-dGMP, c-dAMP, and cGAMP) produced by bacteria are essential for the regulation of bacterial function, such as biofilm formation, colonization, and reproduction (149, 150). As ligands for STING, CDNs can bind directly to and activate STING independently of cGAS, which contributes to several immune responses from bacteria (151). Usually, bacteria can enter or be engulfed by the cell through the endophagosome and be sequestered from the cytosolic sense receptor. Some bacteria, such as *Mycobacterium tuberculosis* (Mtb), can survive in vacuoles, resulting in an insufficient cellular immune response to defend against it (152). In contrast, the ESX-1 secretion system of the mycobacterium can translocate the phagosomal vacuolar matrix including bacterial genome molecules into the cytoplasm and trigger the cGAS-sensing pathway (153). For other bacteria, such as *Legionella pneumophila* or and *Brucella abortus*, the host guanylate binding proteins (GBPs) facilitate rupture of phagosome vacuoles and are indispensable for controlling their infection (154, 155). Autophagy signaling mediated by cGAS/STING is also involved in microorganism clearance mentioned above (90, 91).

Bacteria have evolved strategies to confront this pathway too. Bacterial phosphodiesterase CdnP produced by Mtb or group-B streptococci can degrade CDNs (156, 157). CpsA (a type of Mtb LytR-CpsA-Psr domain-containing protein) can prevent autophagy responses for eliminating pathogens (90). *Chlamydia trachomatis* inclusion membrane proteins can maintain the stability of the inclusion membrane and avoid inclusion lysis (leading to pathogen antigens leaking out and being detected by the host cell) (158, 159). *Yersinia* outer protein J (YopJ) deubiquitinates STING and impedes the formation of the STING signalosome (160). The cGAS-STING pathway activation even impedes the elimination of *Listeria monocytogenes* because bacterial DNA can be packaged into EVs and transferred into T cells, where it induces apoptosis of T cells (161, 162).

Several protozoans, such as *Toxoplasma gondii* and malaria parasites, have an intracellular period in their lifecycle. *T. gondii* could engage cGAS-STING exclusively (163). However, IRF3 activation inducing ISG expression promotes *T. gondii* development independently of IFN expression (163). *P. falciparum* can target erythrocytes, lacking a nucleus and unable produce IFN, but infected erythrocytes can secrete EVs containing parasitic gDNA to monocytes and trigger cGAS (164).

## cGas-Sting Pathway in Autoimmune or Inflammatory Diseases

The immune system is regulated by a complicated network. Disorder of immune signaling can elicit non-infectious inflammatory or autoimmune diseases. Excessive, uncontrolled production of type-I IFN can lead to a spectrum of inflammation diseases termed “type-I interferonopathies,” which have some common manifestations (165). cGAS-STING is the one of main sources of type-I IFN, acts as a cellular immune-sensing signaling axis, and is involved in type-I interferonopathies.

Stimulator of interferon genes -associated vasculopathy with onset in infancy (SAVI) is a typical STING-related hereditary inflammatory type-I interferonopathy, and is manifested by interstitial lung disease, dermatomyositis and arthritis. Its pathology is featured by leukocytoclastic vasculitis and microthrombotic angiopathy of small dermal vessels (166, 167) and patients can also suffer from lymphopenia (166–169). The etiology of SAVI is a gain of function (GOF) mutant in STING which leads to constitutive STING activation without CDNs stimulation (166). Currently, several mutant amino acids residues have been found in or close to the dimerization domain (V155M, N154S, G166E, V147L, and V147M) (64, 166, 168, 170), as well as R284G, R284S, R281Q, and C206Y in the cGAMP-binding domain (171).

Other types of type-I interferonopathies, such as systemic lupus erythematosus (SLE) and Aicardi–Goutières syndrome (AGS), have relationships with defective clearance of cytosolic nucleic acids caused by congenital dysfunction of TREX1, RNASEH2, and SAMHD1. SLE is a heterogeneous autoimmune disease which has prominent type-I and also -II IFN signatures (172). AGS comprises some systemic autoimmune syndromes overlapping with SLE, and can be classified as a “lupus-like disease” (173). Additionally, AGS also causes severe developmental neurological disorders, including cerebral calcifications, encephalopathy and cerebral atrophy.

**FIGURE 4:**
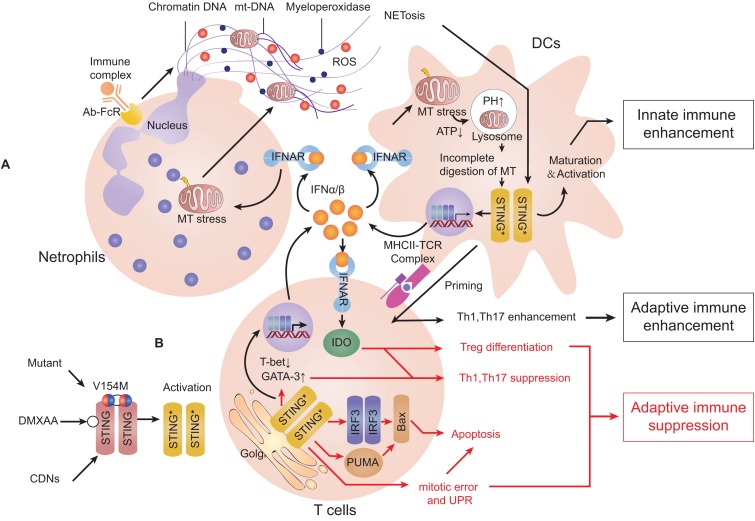
Activation of the cGAS-STING pathway can induce type-I interferonopathies or adaptive immune deficiency. Stimulator of interferon genes (STING) can be stimulated by cyclic dinucleotides (CDNs), agonist compounds and mutants. **(A)** STING activation in dendritic cells (DCs). When exposed to type-I interferon (IFN), neutrophils can release neutrophil extracellular traps (NETs). DCs can internalize self-DNA and trigger the cyclic-GMP-AMP synthase (cGAS)-STING pathway to secrete type-I IFN, which activates DC maturation. Type-I IFN in turn induces mitochondrial stress and decreases ATP production that increases the pH of lysosomes. Incomplete degradation of mitochondria leads to accumulation of mtDNA in the cytosol and activates STING. Mature DCs can present antigen and co-activate signaling to prime T cell-mediated adaptive immune responses. **(B)** STING activation in T cells. IFNα/β enhances differentiation of T helper 2 (T_h_2) cells but suppresses differentiation of T_h_1 cells. Activation of STING can induce mitotic delay and unfolded protein response (UPR), leading to reduced proliferation and promotion of apoptosis. All of the actions stated above suppress the functions of T_h_1 cells and lead to suppression of adaptive immune responses (Red arrows represent mechanisms limiting cellular immune responses, and black arrows represent mechanisms promoting cellular immune responses).

Systemic lupus erythematosus is a representative model for elucidating the mechanism of type-I interferonopathies. In SLE, the level of self-DNA which is packaged into apoptosis-derived membrane vesicles along with the level of anti-dsDNA antibody is increased in the serum of patients (174). A study revealed increasing levels of ISGs (including cGAS/STING) as well as the cGAMP-detected ratio in peripheral-blood mononuclear cells of SLE patients (175). As innate immune cells, DCs have essential roles in antigen presentation, cytokine secretion, and priming the adaptive response of immune cells (176). Plasmacytoid DCs (pDCs) can internalize and recognize self-DNA and they are the main source of type-I IFN in serum during SLE (177). IFNα/β is essential for complete function of immature pDCs (178). IFNα/β and IL-1 can induce mitochondrial oxidative stress in DCs and decrease ATP production, which blocks proton-pump function and increases pH of lysosomes. This process inhibits mitochondrial degradation and blocks mtDNA clearance, which engages the cGAS-STING pathway (31, 179). Moreover, monocytes may sense mtDNA through cGAS-STING and differentiate into DCs (31, 180). Neutrophil extracellular traps (NETs) are complexes released by neutrophils exposed to stimuli or autoantibody immune complexes. NETs comprise extracellularly released chromatin, myeloperoxidase enzymes, and also oxidized mtDNA. In lupus-like diseases, NETs can be induced by IFNα/β and may play a major part in priming pDCs (181, 182). All mechanisms stated above contribute to a more aggravated type-IIFN response and exacerbate disease. A similar phenomenon can be observed in SAVI, ataxia telangiectasia (AT) and Artemis deficiency (183). However, compared with SAVI, DCs in SLE can prime T-cell maturation significantly and increasing secretion of pro-inflammatory cytokines, such as IL-6 and TNFα can also lead to activation of adaptive immunity (Figure 4A).

The cGAS-STING pathway can mediate systemic inflammation as well as autoimmune activation. However, it is also involved in the local inflammation of multiple tissues. With regard to ischemic myocardial infarction (MI), cardiac macrophages can sense dying ruptured cells and lead to fatal post-MI cardiac inflammation, which is reversed by ablation of cGAS/STING/IRF3 (184, 185). In a non-alcoholic steatohepatitis (NASH) model induced by a methionine- and choline-deficient or high-fat diet, lipotoxicity can cause mitochondrial damage and up-regulate STING/IRF3 expression in hepatocytes, which in turn promotes lipid accumulation and inhibits glycogen synthesis. All above bring out hepatic inflammation and hepatocytes apoptosis (186). In this model, mice with deficiency of STING presents alleviated insulin resistance and lower levels of low-density lipoprotein in serum, and also decreased hepatic inflammation and fibrosis/steatosis, in which hepatic macrophages/kupffer cells may take a big part (187, 188). Lipotoxicity can induce p62 to be phosphorylated through the cGAS-STING-TBK1 pathway, which causes aggravated protein inclusions in hepatocytes and it indicates that p62 could be a biomarker for NASH prognosis (189). mtDNA-dependent inflammation induced by lipotoxicity also occurs in adipose tissue and endothelial cells of blood vessels, which contributes to tissue inflammation, insulin resistance, and cardiovascular diseases (42, 190). In traumatic brain injury (TBI), local injury initiates breakdown of the blood–brain barrier and global neuroinflammation (191). STING expression is up-regulated in TBI and can lead to increased expression of pro-inflammatory cytokines and enlargement of secondary injury (192). Reduced autophagy-associated protein expression induced by STING may contribute to the dysfunction of autophagy and dampen the elimination of necrotic tissue, thereby intensifying inflammation (192). During silicosis, silica can yield cytosolic dsDNA release and engage cGAS-STING, which activates DCs and macrophages to cause severe lung inflammation. It also leads to death of epithelial cells through the NLRP3 pathway and pulmonary fibrosis (193). Similarly, mtDNA release in renal tubule cells has been found to be associated with acute kidney injury by cytotoxic drugs and chronic renal fibrosis (194, 195).

Neurodegenerative diseases are correlated with local inflammation (196). In the central nervous system (CNS), microglia is considered to be the main source of cGAS-STING-dependent IFN expression (197). In neurodegenerative diseases, levels of the marker of microglia activation-cluster of differentiation 68 (CD68), and pro-inflammatory cytokines are increased (198). A significant feature of Parkinson’s disease (PD) is the neuronal loss of cerebral nuclei (especially dopaminergic neurons in the substantianigra). Serine/threonine-protein kinase PINK1 and E3 ubiquitin-protein ligase parkin are ubiquitin-related factors that take part in removing damaged mitochondria by autophagy, and their dysfunction lead to the early onset of PD (199). Parkin^–/–^ and PINK1^–/–^ mice, following exhaustive exercise, show inflammation and loss of dopaminergic neurons, which can be rescued by loss of STING (200). Similarly, AT is a genetic disease caused by missense mutation of a DNA-repair protein: ATM. AT patients usually show neurodegenerative defects (especially ataxia) complicated with telangiectasia on their eyes or body, deficiency of adaptive immune cells, and predisposition to cancer (201). Nevertheless, up-regulation of expression of type-I IFN can also be found in AT patients and mice, causing them to be prone to autoimmune diseases (36, 183, 202). This syndrome is related to p53-mediated senescence but also the chronic inflammation mediated by the cGAS-STING pathway which engages cytosolic uncombined broken gDNA caused by ATM dysfunction (127). In addition, accumulation of cellular mtDNA occurs in age-related macular degeneration characterized by retinal pigmented epithelium (RPE) degeneration. This can trigger chronic inflammation by cGAS-STING pathway, in which NLRP3 inflammasomes, inflammatory/apoptotic caspases are also involved (203, 204).

With regard to other diseases in which adaptive immune cells prime, cGAS-STING has a different role. Multiple sclerosis (MS) is a local inflammatory disease of CNS. MS is characterized by over-reactive microglia, infiltration of self-reactive T cells, demyelization of nerve fibers and hyperplasia of gliocytes. Autoantibodies against proteins expressed in immune-privileged regions of CNS also contribute to its pathogenesis (205). In MS, IFNα/β can attenuate disease severity effectively. This implies a protective role for type-I IFN in CNS, which is considered to counteract the pro-inflammatory IFNγ (206). Using experimental autoimmune encephalitis (EAE) as a MS model, STING was found to be indispensable for amelioration of type-I IFN-mediated neuroinflammation, and it could be induced by a conventional anti-viral drug ganciclovir (207). Ultraviolet (UV) radiation is a factor inversely related to the morbidity of MS (208). It was found that UV-B irradiation can recruit inflammatory monocytes and produce type-I IFN in a STING-dependent manner (209). All above indicate that cGAS-STING-IFNα/β pathway may have a beneficial effect on some CNS inflammatory diseases such as MS.

A possible reason for the observed effect above is due to indoleamine 2,3-dioxygenase (IDO), which can catabolize tryptophan (Trp) oxidatively. Trp withdrawal or Trpoxidative catabolites can interact with general control non-derepressible 2 (GCN2) and mTOR, of which both can control cellular amino-acid metabolism and suppress T helper 1 (T_h_1) cells immunity (210). IFNα/β is a potent IDO inducer to suppress proliferation of CD4^+^ T_h_1 cells and promote differentiation of Foxp3^+^ regulatory T (T_reg_) cells, which are believed to suppress CNS-specific autoimmunity (210, 211). In addition, DNA released from dying cells can be internalized directly by T cells and sensed by cGAS-STING pathway, which leads to enhancement of the T_h_2 transcription factor GATA3 but suppression of the T_h_1 transcription factor T-bet. Consequently, this process polarizes naive T cells toward T_h_2 differentiation (212). Studies mentioned above may (at least in part) explain why the cGAS-STING signal is a negative regulator of MS.

The inhibitory role of cGAS-STING in inflammation is also attributed to its apoptosis-triggering role. In some subtypes of SAVI and mouse models, apoptosis of blood-vessel endothelial cells or bronchial epithelial cells and leucopenia can be observed (especially T-cell lymphopenia) (166, 169, 170). When the STING signal is stimulated, apoptosis occurs more frequently in normal or cancerous T cells (119). Also, bone-marrow chimeras and gene-knockout studies have shown that T cells defect in SAVI are not associated with type-I IFN signaling or cGAS (213, 214). Localization of STING at the Golgi can cause delay of T-cell mitosis and reduced proliferation independently of IRF3 and TBK1 (215). Furthermore, a “UPR motif” on the C-terminus of STING can cause ER stress and UPR, resulting in Ca^2+^ overloading and T-cell death (82). A controversial view is that B cells express STING variously and may undergo apoptosis through this way (166, 216, 217). However, simultaneous signaling by STING and the B-cell antigen receptor can promote B-cell activation and antibody production independently of type-I IFN (217) (Figure 4B).

As for some diseases with inflammatory responses involved, the acute phase of pancreatitis causes dying acinar cells to produce free dsDNA, which activates cGAS-STING signaling in macrophages, and exacerbates inflammation severity (218). However, in the chronic phase of pancreatitis, cGAS-STING activation decreases pancreatic inflammation, which may be mediated by limiting T_h_17 response (219). For gut mucosal immunity, transient stimulation of STING could strengthen the function of antigen-presenting cells (APCs) and promote T_h_1 and T_h_17 immune responses against microbes (220). Chronic STING signaling, however, elicits an IL-10 response to control the inflammation and avoids inflammatory enterocolitis such as bowel disease (221). STING knockout mice present reduced numbers of goblet cells, a decreased ratio of commensal versus harmful bacteria and compromised T_reg_ cells in the gut, making it prone to enterocolitis (222).

## cGas-Sting Pathway in Cancer

Chromosomal instability (CIN) is an intrinsic feature of cancer, and results spontaneously from errors in chromosome segregation during the mitosis of cancer cells. CIN can also be induced manually by radiotherapy or chemotherapy, which causes DSBs. It results in micronuclei formation outside the nucleus, of which rupture brings out irrepressible accumulation of cytosolic self-DNA and engages cGAS (32, 38, 223). However, normal mitotic processes involve exposure of chromosomal DNA to the cytoplasm, but this cannot initiate a substantial inflammatory reaction or apoptosis because nucleosomes can suppress dsDNA-cGAS binding in a competitive manner (41).

An appropriate immune response against tumors *via* a type-I IFN plays an indispensable part in limiting tumors and prolonging host survival (224). It was found STING-deficient mice are prone to developing several types of cancer and have poor survival under a tumor burden, whereas stimulation of STING can elicit robust immunity to tumors (225–227). A mechanism is many cancer cells expressing cGAS can recognize cytosolic DNA and produce cGAMP to stimulate secretion of type-I IFN through STING (228, 229). Excessive expression of TREX1 in cancer cells, which can be induced by radiotherapy, attenuates this progression (228). cGAS-STING can also promote senescence of cancer cells through the p53-p21 pathway (129). cGAS-STING-mediated autophagy contributes to autophagic cell death if mitotic crisis occurs to avoid transformation of cancer cells (121). Melanoma cells can also transfer cGAMP produced by them to proximal non-cancerous host cells through gap-junction channels and activate STING in these cells, which contributes to the recruitment of tumor-infiltrating immune cells such as natural killer (NK) cells (51, 230). Expression of the NK cell-specific ligand NKG2D retinoic acid early transcript 1 (RAE1) on cancer cells is highly up-regulated by STING once NK cells permeate into tumor tissue (231). The activation of STING in the endothelium within the tumor microenvironment (TME) could contribute to the remodeling of tumor vasculatures, and may have positive effects on tumor regression (232).

Dendritic Cells are the main source of type-I IFN in several types of TMEs and are dependent on STING signaling (229). More preferentially than macrophages, infiltrating DCs take up tumor-derived DNA or cGAMP from dying cell fragments by phagocytosis (27, 29, 129, 226, 233). Moreover, cancer cells can package DNA into exosomes and transfer DNA to DCs (234). Produced cGAMP by cancer cells can also be transferred to DCs through forming gap junction (53). By activating cGAS-STING signal in DCs, CD8α^+^ subtype DCs secret chemokines such as CCL5 and CXCL10 and cross-prime infiltrating anti-tumor CD8^+^ T cells (29, 226, 235–237). In contrast, numbers of immune-suppressing cells such as T_reg_ cells, myeloid-derived suppressor monocytes and M2 macrophages have been reported to be decreased (225, 238, 239). Expression of IL-15/IL-15Rα complex is up-regulated in myeloid cells with the help of STING/type-I IFN and promotes tumor regression (240).

Tumor cells can evade intrinsic cellular surveillance in different ways. In various cancer cell lines, cGAS, STING, TBK1, and IRF3 are mutated frequently and their decreased expressions are also related to the high level of methylation (241). STING expression has been shown to be suppressed by the alternative lengthening of telomeres (ALT) pathway, which is responsible for prolonging the telomere length and maintaining the proliferation of tumor cells (242). A hypoxic environment in tumor cells can lead to accumulation of lactic acid and is associated with the inhibition of tumor-conditional DCs and reduced expression of IFN signaling molecules (243). In breast cancer, functional up-regulation of expression of human epidermal growth factor receptor 2 (HER2), a ligand-independent receptor tyrosine kinase (RTK), can arrest the expression of RAC-alpha serine/threonine-protein kinase (AKT1) (a key factor in the mTOR pathway), which is reported to inhibit the activation of cGAS and TBK1 (244, 245).

Patients with lung adenocarcinoma have a low probability of survival if they have reduced expression of cGAS (132). Thus, expression of the cGAS-STING and DNA-damage marker histone γH2AX in tumor cells could be considered as independent prognostic factors to predict therapy response and clinical outcome, and could be superior to that of traditional markers like immunogenic cell death and T cells number (246).

However, some scholars have arrived at opposite conclusions. When DSBs occur in cancer cells, cGAS can be relocated to the nucleus and obstruct the formation of the PARP1-Timeless complex, thereby inhibiting homologous recombination repair and maintaining CIN, which potentiates tumor evolution (35, 223). It has also been reported that cGAS recognizing CIN activates non-canonical NF-κB signaling and potentiates cellular metastasis programs (247). Furthermore, STING^–/–^ mice are resistant to skin carcinogenesis in a 7,12-dimethylbenz(a)anthracene (DMBA)-treatment model. It has been demonstrated that when DMBA-induced nuclear DNA leaks into the cytoplasm, STING can induce chronic inflammatory stimulation that contributes to cancer development (248). During brain metastasis, cGAMP transferred to bystander cells (e.g., astrocytes) can also produce IFNα and TNFα in the TME but, in this context, it will support tumor development and chemoresistance (249). Coordinating with myeloid cells penetrating into the tumor, myeloid-derived suppressor cells can also be recruited through the C-C chemokine receptor type 2 (CCR2) (250). Another study found that microparticles yielded by tumor cells can turn macrophages into the M2 type through cGAS-STING-TBK1, contrary to previous findings (251).

**FIGURE 5:**
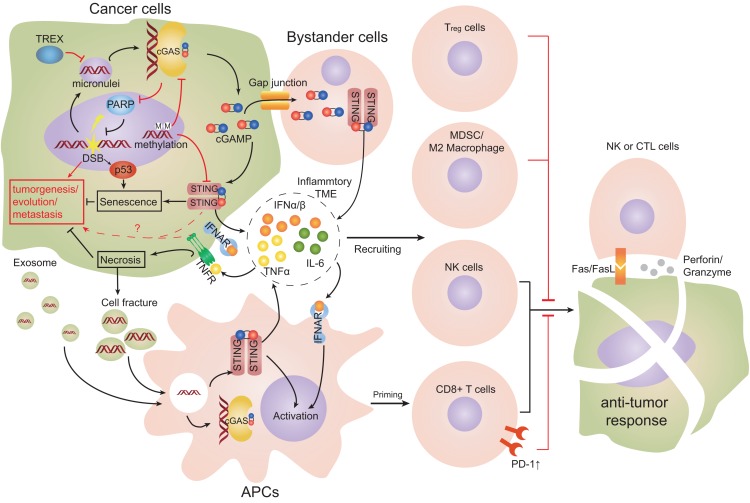
Cell interactions in TME engaging the cGAS-STING pathway. Double-stranded breaks (DSBs) can lead to cytosolic self-DNA that engages the cyclic-GMP-AMP synthase (cGAS)-stimulator of interferon genes (STING) pathway and produces type-I interferon (IFN). STING promotes the senescence of cancer cells. Cyclic guanosine monophosphate-adenosine monophosphate (cGAMP) can transfer to proximal host non-cancer cells through gap-junction channels and promote the formation of an inflammatory tumor microenvironment (TME). Dendritic cells (DCs) are the main source of type-I IFN and tumor necrosis factor α (TNFα), and promote an inflammatory TME. Infiltrating DCs take up tumor-derived DNA or cGAMP from dying cell fragments or exosomes. DCs cross-prime and activate anti-tumor cluster of differentiation 8 (CD8)^+^ T cells and constrict cancer growth effectively. Co-stimulation of type-I IFN and TNFα signaling simultaneously leads to remarkable necroptosis of tumor cells. cGAS can relocate to the nucleus and obstruct polymerase 1 (PARP1), which suppresses DNA repair. Chronic levels of IFNα and TNFα in TME could support tumor development and cause type-2 macrophages and regulatory T cells to be recruited (Red arrows represent mechanisms promoting tumors; black arrows represent mechanisms limiting tumors).

Immune-system interactions with tumor cells are complicated. The effect of cGAS-STING on cancer is dependent on the type of tumor, host immune state, activated cell types, therapeutic intervention, and the magnitude of cGAS-STING activation. Like inflammation generated by cGAS-STING, a time-dependent inflammatory anti-tumor response mediated by cGAS-STING may be present. Temporary activation of cGAS-STING in innate immune cells could enhance the anti-tumor effect, whereas sustained activation of cGAS-STING might induce immune tolerance of the tumor. More investigations are necessary to ascertain the exact role of cGAS-STING in oncology, and elucidate the specific advantages and adverse effects of targeting the cGAS-STING pathway in cancer therapy (Figure 5).

## Targeting the cGas-Sting Pathway for Treatment

Considering the pivotal role of the cGAS-STING pathway in infection, inflammation and cancer, positive modulation of the pathway signaling is a promising way to enhance the immune state and restrict microorganisms or heterogeneous cells, whereas negative modulation can control aberrant inflammation.

Radiotherapy or chemotherapy drugs such as cisplatin or cyclophosphamide can induce DSBs and micronuclei, then trigger the cGAS-STING pathway to enhance tumor immunogenicity (252–254). In addition, PARP inhibitors such as olaparib have promising effects on cancer cells lacking BRCA2 because of their cooperative DNA-repair functions (253). Although cGAS activation is inhibited by nucleosomes, taxol can induce mitotic cell-cycle arrest and sustain divided chromatin in the cytosol to activate the cGAS-STING pathway slowly, and accumulation of signaling could stimulate apoptosis of cancer cells (41). Inhibitors of topoisomerase 1 or 2 used conventionally as chemotherapy drugs trigger minor damage to DNA and accumulation of cytosolic DNA, which can engage cGAS and enhance the anti-tumor or anti-infection responses of cells (255–257).

cGAS-STING is essential on anti-tumor immune checkpoints therapies. For example, blockade of CD47-signal regulatory protein α (SIRPα) signaling on DCs can activate NADPH oxidase 2 (NOX2) and increase the pH in phagosomes along with incomplete degradation of mtDNA, which can trigger cGAS-STING (129). STING deficiency in mice abrogates the anti-tumor effect of CD47 blockade (258). A similar phenomenon also can be seen in anti-programmed cell death-1 (PD-1) therapy (259). There is greater infiltration of IFNγ^+^ cells and CD8^+^ T cells and PD-1/PD-1 ligand 1 (PD-L1) expression in TME treated by STING-ligand derivatives (260). Therefore, in several types of tumors, combined administration of a STING agonist and immune-checkpoint antibody could elicit a more curative outcome compared with one therapy alone (238, 261).

Viruses can infect cells lacking cGAS-STING more effectively, and have higher oncolytic activity compared with virus therapy alone. Hence, the use of oncolytic viruses such as talimogene laherparepvec is beneficial for treating tumors with low expression of cGAS/STING. STING expression can be regarded as a prognostic measurement for such therapy (262).

Some artificial analog molecules of CDNs, such as 5,6-dimethylxanthenone-4-acetic acid (DMXAA) and 10-carboxymethyl-9 (10H) acridone (CMA) can bind the CDN pocket of mouse-specific STING dimers and promote conformational transition of STING from inactive “open” to an active “closed” state (263, 264). DMXAA showed convincing efficacy in restraining tumors in mice (265). However, DMXAA is restricted in activating mouse-specific STING but not human-specific STING, which could be an explanation for the failure of DMXAA in treating 1299 non-small-cell lung cancer patients in a phase-III clinical trial (NCT00662597) (266). Nonetheless, with three substitutions (G230I, Q266I, and S162A), human STING can also be induced by DMXAA to undergo conformational transition (264). Another new compound, amidobenzimidazole, has been found to be an agonist of STING without lid closure and has potential therapeutic value (267).

Cyclic dinucleotides and their derivates that can stimulate STING directly are candidate adjuvants to restrain tumors. Intratumoral administration of c-dAMP, c-dGMP, or cGAMP analogs alone or combined with other adjuvants or tumor antigens have shown anti-tumor effects (259, 268); phase-I or II clinical trials (NCT02675439, NCT03172936, and NCT03937141) of dithio-(RP,RP)-c-dAMP (known as ADU-S100) are ongoing (261). To avoid the degradation and ensure maximal phagocyte internalization of CDNs, endosomolytic nanoparticles have been designed to package and deliver CDNs. For example, pH-sensitive nanoparticles (e.g., STING-nanoparticles) can release their contents if located in acidic endosomal environments (269).

For treatment of type-I interferonopathies, lessons can be taken from the treatment of canonical autoimmune disease such as SLE, but there are several differences. For example, it was found that corticosteroid pulse therapy, γ-immunoglobulins, disease-modifying anti-rheumatic drugs, anti-CD20, and some immunosuppressants (e.g., methotrexate) have limited efficacy against SAVI (166, 171). JAK inhibitors such as ruxolitinib, tofacitinib and baricitinib that reduce type-I IFN downstream signaling have shown therapeutic value against SAVI, but further verification of their efficacy is needed (270). Moreover, novel immune therapies, such as anti-IFNα and anti-IFNAR immunoglobin, are in clinical trials for SLE. These could also be tested against SAVI in the future (165).

Pharmaceutically screening has revealed that some anti-malaria drugs, such as suramin, have an inhibitory effect on cGAS by blockade of interaction between DNA and cGAS (271). In addition, novel small molecules such as RU320521 or G150 can occupy the enzymatic pocket of species-specific cGAS to abrogate cGAMP synthesis (272, 273). Recently, a study found that aspirin can acetylate cGAS at three lysine residues and block cGAS activity (274). With regard to STING, the cyclopeptide Astin C can block IRF3 recruitment onto the STING signalosome (275). The molecule H-151 can block the palmitoylation of human-STING (276). All of these agents are potential candidates for alleviating type-I interferonopathies.

## Concluding Remarks and Future Perspectives

The cGAS-STING pathway is primarily responsible for the modulation of immune response in cells when facing cytosolic dsDNA challenge. Moreover, it is complicatedly cross-regulated by other cellular processes or cellular signaling networks. The exact fundamental mechanism of the pathway in cells and the effect on the whole organism in specific states is not completely clear and requires further investigation.

In conclusion, the cGAS-STING pathway has dichotomous roles in the immune system. In general, cGAS-STING-type-I IFN signaling can promote the innate immune response in myeloid cells but alleviate the adaptive immune response exerted by T cells and B cells. cGAS shows high expression in APCs such as macrophages and DCs, but STING is expressed in most cells (10). cGAS-STING signaling corresponding to cytoplasmic dsDNA in APCs can boost innate and adaptive immunity transiently. In this situation, the DNA challenge signal is limited to only macrophages and DCs. Their pro-inflammatory and antigen-presenting functions to adaptive immune cells are promoted in the short-term. If the signal spreads to other bystander cells, such as T cells, B cells, local resident cells by means of cGAMP transfer, or just aberrant STING activation by GOF, it causes apoptosis in bystander cells or adaptive immune cells and immune tolerance in the long-term. Therefore, it is reasonable to conclude that the intrinsic function of the cGAS-STING pathway is essential for the innate immune system responses of the host immediately after pathogen invasion or abnormal cell appearing. Once the challenge persists, the cGAS-STING pathway controls the adaptive immune system to avoid chronic, detrimental inflammatory reactions or autoimmune diseases.

The inflammatory response exists universally in almost all physiologic and pathologic progressions. cGAS-STING is pivotal in modulating cellular inflammation, so it is promising to extend our conception of the cGAS-STING pathway onto more diseases with inflammatory responses involved, especially CNS-based diseases such as stroke, in which the inflammatory reaction exists but was recognized less.

Moreover, for targeting the cGAS-STING pathway for therapeutic purposes, drugs should be optimized to augment the desirable effect and prevent its unwanted effects. For example, to eliminate tumor cells or infectious agents, agonists of cGAS-STING would be a rational option if designed to target APCs exclusively but not T cells or B cells. In this scheme, the anti-tumor immune response is enhanced while avoiding apoptosis of adaptive immune cells and infiltration of immune suppressor cells. Also, most research on the cGAS-STING pathway has focused on its IFN-expressing role but overlooked autophagy and cell-death roles, which are also main downstream signaling of the pathway. Therefore, some drugs, such as emricasan, are potential apoptosis inhibitors that may have a complementary effect on ameliorating apoptosis of blood-vessel endothelial cells or bronchial epithelial cells, and lymphopenia, in SAVI.

Until now, studies of the cGAS-STING pathway have been done mainly in the laboratory but it has large space to be explored in clinical or translational fields. Additionally More PRRs and cellular immune-surveillance pathways may remain to be discovered to piece together the molecular puzzles of the cell.

## Author Contributions

DW drafted the manuscript and drew the figures. WJ and JH conceived and revised the review.

## Conflict of Interest

The authors declare that the research was conducted in the absence of any commercial or financial relationships that could be construed as a potential conflict of interest.
